# Large-scale single-cell cloning of genome-edited cultured human cells by On-chip SPiS

**DOI:** 10.1016/j.xpro.2023.102364

**Published:** 2023-06-16

**Authors:** Gou Takahashi, Yuichiro Miyaoka

**Affiliations:** 1 Regenerative Medicine Project, Tokyo Metropolitan Institute of Medical Science, Tokyo 156-8506, Japan; 2 Graduate School of Medical and Dental Sciences, Tokyo Medical and Dental University, Tokyo 113-8510, Japan; 3 Graduate School of Humanities and Sciences, Ochanomizu University, Tokyo 112-8610, Japan

**Keywords:** Cell Biology, Cell Isolation, Single Cell, Molecular Biology, CRISPR, Biotechnology and Bioengineering

## Abstract

Single-cell cloning is the simplest strategy to isolate genome-edited cell clones, although its scalability has been an issue. Here, we present a protocol to establish genome-edited human cultured cell clones using the On-chip SPiS, a single-cell auto-dispensing device with image recognition technology. Human cultured cells are transfected with plasmids of the CRISPR-Cas9 components, and Cas9-expressing cells are sorted and individually plated into multi-well plates by the On-chip SPiS.

For complete details on the use and execution of this protocol, please refer to Takahashi et al. (2022).^1^

## Highlights

• A protocol for efficient genome-edited cultured cell clone establishment is shown
• On-chip Sort is used for simple and rapid isolation of cells expressing Cas9
• SPiS implements automated single-cell dispensing by image recognition
• Over 80 genome-edited human cell clones can be isolated in one experiment

## Abstract (editor highlights)

Publisher’s note: Undertaking any experimental protocol requires adherence to local institutional guidelines for laboratory safety and ethics.

## Graphical abstract

**Figure:**
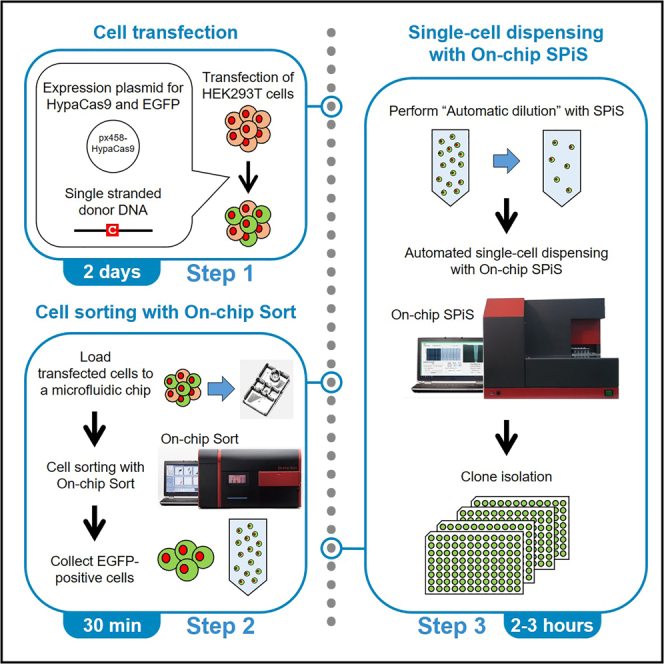

## Before you begin

The flexible genetic engineering made possible by genome editing is widely used in basic biology, agriculture, industry, and medicine.^2^^,^^3^^,^^4^ DNA double-strand break-mediated genome editing systems cleave target sequences in the genomic DNA, leading to activation of the DNA repair pathways, including non-homologous end joining (NHEJ) and homology-directed repair (HDR).^5^^,^^6^ The resulting cells have various genotypes, and single-cell cloning is the most direct way to identify the genotypes of these cells individually. In this protocol, we describe the single-cell cloning process of HEK293T cells whose *RBM20* gene has been edited to introduce a single-nucleotide substitution, as we previously reported as an example.^7^ We use HypaCas9 because it increases the HDR frequency, as we previously described.^8^ We have confirmed that this protocol works with HeLa cells and PC9 cells as well and have established more than 2,600 clones of these cultured human cells using the single particle isolation system (SPiS).^1^ This protocol may also be applicable to Base Editing^9^^,^^10^^,^^11^ and Prime Editing.^12^

In this protocol, we use pX458-HypaCas9 (Addgene plasmid #190604) to express EGFP via the T2A peptide fused to HypaCas9 to select only cells that expressed Cas9. EGFP-positive cells were sorted with a microfluidic cell sorter (On-Chip Sort^13^) and then dispensed individually with the SPiS. Using this protocol, we can establish more than 80 genome-edited clones in a one-time experiment.

### Preparation of plasmid DNA

**Timing: 3–5 days**

This section describes the design of gRNAs and short single-stranded donor DNAs for genome editing.

**Figure 1:**
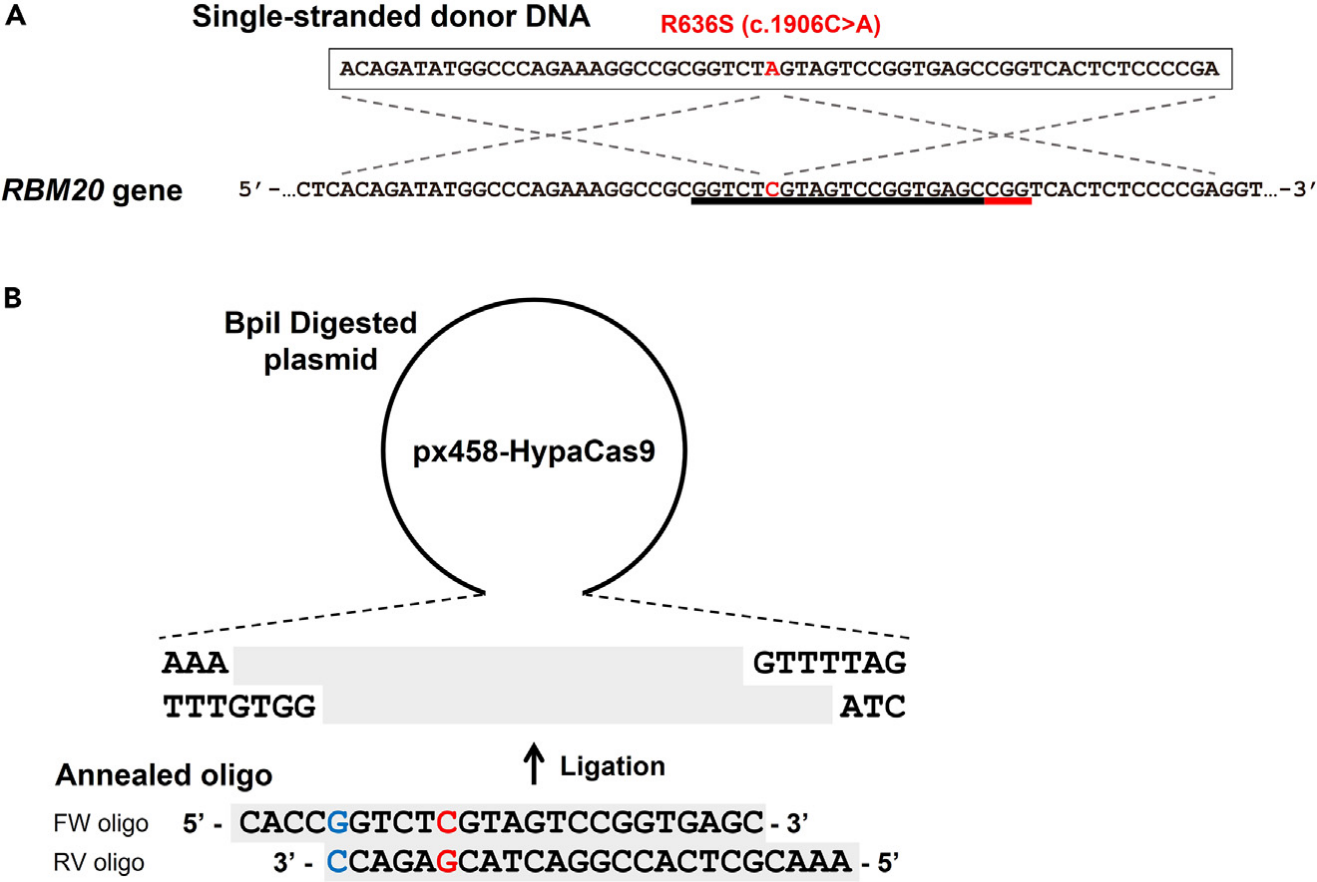
Design of donor DNA and cloning of a gRNA into px458-HypaCas9 for editing *RBM20* (A) Design of single-stranded donor DNA for R636S (c.1906C>A) point mutagenesis in *RBM20*. Red nucleotides indicate the substituted nucleotides. The black and red underlines indicate the gRNA and PAM sequences, respectively. (B) Cloning of a gRNA for point mutagenesis. A cytosine (red) was substituted, and a 5′ guanine (blue) of the annealed 20-bp oligonucleotides was required for optimal transcription driven by the human U6 promoter.

1. Design of gRNAs and single-stranded donor DNAs for single nucleotide substitutions.
  a. Design a gRNA using Cas-Designer^14^ (http://www.rgenome.net/cas-designer/) or CRISPRdirect^15^ (http://crispr.dbcls.jp/) in a gene of interest.
    ***Note:*** The cut site should be within 10 bp from the single-nucleotide substitution. It is also desirable to locate the nucleotide substitutions within the gRNA or PAM sequence to avoid re-cleavage of edited alleles by Cas9.
    ***Note:*** If there is no cut site available for SpCas9, the Cas9 orthologs or engineered Cas9 variants with different PAM sequences from SpCas9 can be used (e.g., SaCas9,^16^ SpCas9-NG^17^).
  b. The single-stranded donor DNA should be 60 nucleotides (nt) in length with the single-nucleotide substitution in the middle (5′-29-nt homologous sequence + 1-nt substitution + 30-nt homologous sequence-3′) (Figure 1A). The 60-nt single-stranded donor DNA can induce substitutions of up to 2 nucleotides. We recommend to extend the single-stranded donor DNA length when substitutions of 3 nucleotides or more are intended.
    ***Alternatives:*** Other gRNA design software programs can also be used (e.g., CHOPCHOP^18^ [http://chopchop.cbu.uib.no/] and Benchling [https://www.benchling.com/crispr]).
    ***Note:*** The human U6 Promoter in px458-HypaCas9 requires a guanine (G) base to initiate efficient transcription (Figure 1B). If the sequence does not begin with a G, add a G at the 5′ end to make a 21-nt gRNA. The purification grade of oligonucleotides for both donor and gRNA is reversed-phase chromatography (RPC).

2. Cloning of gRNAs.
  ***Note:*** Our protocol for gRNA cloning into the px458-HypaCas9 plasmid is a slightly modified version of the one used for pX330-based plasmids established by Dr. Feng Zhang (https://www.addgene.org/crispr/zhang/#spcas9).^19^ The original protocol can be applied here too.
  a. Digestion and dephosphorylation of px458-HypaCas9.
    i. Order px458-HypaCas9 (#190604) from Addgene.
    ii. px458-HypaCas9 is digested by BpiI (BbsI) and dephosphorylated by Fast AP (alkaline phosphatase) as shown in below Table.
      **Table:** Restriction and dephosphorylation solution.

Reagent	Final concentration	Amount
px458-HypaCas9 plasmid (1 μg/μL)	50 ng/μL	1.0 μL
FastDigest BpiI	1x	1.0 μL
FastAP	1 unit/20 μL	1.0 μL
FastDigest Green Buffer (10x)	1x	2.0 μL
MQW	N/A	15.0 μL
**Total**	**N/A**	**20.0 μL**
    iii. After running the digested plasmid on a 1% agarose gel electrophoresis, the band is cut out, and the DNA is purified with a common gel purification kit (e.g., FastGene Gel/PCR Extraction Kit), and eluted with 20 μL of MQW. The DNA concentration should be around 20 ng/μL.
  b. Phosphorylation, annealing, and ligation of oligonucleotides for gRNAs.
    i. For one gRNA, order two complementary oligonucleotides with the gRNA sequence and 5′ overhangs: a FW oligonucleotide with a 5′-CACC (or 5′-CACCG if a guanine is needed) overhang and an RV oligonucleotide with a 5′-AAAC (Figure 1B).
    ii. Prepare the T4 PNK (T4 polynucleotide kinase) and oligonucleotides mixture shown in below Table.
      **Table:** Annealing and phosphorylation solution

Reagent	Final concentration	Amount
FW oligonucleotide (100 μM)	10 μM	1.0 μL
RV oligonucleotide (100 μM)	10 μM	1.0 μL
T4 DNA Ligase Reaction Buffer	1x	1.0 μL
T4 PNK	5 unit/10 μL	0.5 μL
MQW	N/A	6.5 μL
**Total**	**N/A**	**10.0 μL**
    iii. Phosphorylate and then anneal two oligonucleotides by incubating the mixture at 37°C for 30 min and at 95°C for 5 min and then ramping down to 25°C by 5 °C/min using a thermocycler.
    iv. Dilute the reaction solution by 250 with MQW.
    v. Prepare the ligation solution, including Ligation High ver.2 (T4 DNA ligase) in a 1.5-mL tube as shown in below Table to clone the annealed oligonucleotides into the linearized plasmid.
      **Table:** Ligation reaction solution

Reagent	Final concentration	Amount
Annealed oligonucleotides (diluted by 250x)	0.1 μM	0.5 μL
Linearized Plasmid	10 ng/μL	0.5 μL
Ligation High ver.2	1x	1.0 μL
**Total**	**N/A**	**2.0 μL**
    vi. Incubate at room temperature for 30 min, and then place the tube on ice.
  c. Transformation.
    i. Add 2.0 μL of the ligation reaction to 10 μL of chemically competent cells (preferably DH5-Alpha) in a 1.5-mL tube.
    ii. Place the tube on ice for 10 min.
    iii. Place the tube in a 37°C water bath for 40 s for heat-shock and then on ice for 1 min.
    iv. Add 750 μL of SOC medium and pre-culture the Escherichia coli at 37°C for 30 min.
    v. Plate the transformed E. coli on LB-ampicillin (100 μg/mL) plates and incubate at 37°C overnight (12–20 h).
    vi. Pick a few colonies and grow at 37°C overnight (12–20 h) in 1.5 mL of LB-ampicillin (100 μg/mL) liquid culture.
    vii. Purify the plasmid by minipreps using a commercial kit of your choice.
      ***Note:*** We recommend a transfection-grade plasmid purification kit, such as the Zyppy Plasmid Miniprep Kit.
    viii. Measure the DNA concentration and quality spectrophotometrically and confirm the sequences by Sanger sequencing. Primers for PCR amplification and Sanger sequencing of the *RBM20* target site in this protocol are shown in the key resources table.

### Cell maintenance and preparation of conditioned medium

**Timing: 2 weeks**

In this section we describe how we acclimate and maintain HEK293T cells and prepare conditioned medium, which supports the cell survival after single-cell dispensing. We also recommend adding antibiotics to the cell culture. Penicillin/Streptomycin (Pen/Strep) is an antibiotic reagent commonly used for cell culture and avoids contamination during the cell sorting and single-cell dispensing steps in this protocol.

1. Thawing and maintenance of cells.
  a. Thaw a frozen HEK293T cell vial (we freeze approximately 2×10^6^ cells per vial) in a 37°C water bath.
  b. Mix the thawed cells and 10 mL of PBS in a 15-mL tube.
  c. Centrifuge at 280 × *g* for 3 min at room temperature.
  d. Remove supernatant with an aspirator.
  e. Resuspend cells in 10 mL growth medium, and then transfer them to a 10-cm dish.
  f. Once cells reach confluence, passage the cells at a 1:20 ratio.

***Note:*** HEK293T cells typically require a passaging in 4–5 days. No medium change is required between passages.

2. Preparation of conditioned medium.
  ***Note:*** This protocol utilizes conditioned medium to enhance the cell survival after single-cell dispensing. Conditioned medium (10 mL) is required for each 96-well plate.
  a. Remove the growth medium and wash the cells once with 10 mL PBS.
  b. Add 1 mL 0.25% Trypsin-EDTA to confluent HEK293T cells in a 10-cm dish and incubate at 37°C for 3 min.
  c. Add 9 mL of growth medium and resuspend the cells.
  d. Transfer 0.5 mL of the cell suspension to a new 10-cm dish and add 10 mL of new growth medium and mix.
  e. Prepare multiple dishes depending on the number of 96-well plates for single-cell dispensing. You will need 10 mL of conditioned medium (the amount you can obtain from a 10-cm dish) per 96-well plate.
    ***Note:*** You can use larger culture plates to prepare conditioned medium.
  f. After 3–5 days of culture, collect the medium as conditioned medium in a 50-mL tube.
  g. To avoid contamination of cell debris, centrifuge the tube at 280 × *g* for 3 min, and then transfer the medium in a new 50-mL tube by pipetting. Conditioned medium can be stored at 4°C for up to 2 months.

## Key resources table

**Table:** 

REAGENT or RESOURCE	SOURCE	IDENTIFIER
**Chemicals, peptides, and recombinant proteins**		

FastDigest BpiI	Thermo Fisher Scientific	Cat# FD1014
T4 DNA Ligase Reaction Buffer	New England Biolabs	Cat# B0202
T4 Polynucleotide Kinase (T4 PNK)	New England Biolabs	Cat# M0201
Ligation High ver.2	Toyobo	Cat# LGK-201
FastAP Thermosensitive Alkaline Phosphatase	Thermo Fisher Scientific	Cat# EF0652
FastDigest Green Buffer (10X)	Thermo Fisher Scientific	Cat# B72
Dulbecco’s modified Eagle medium (DMEM) with high glucose, sodium pyruvate, and L-glutamine	Thermo Fisher Scientific	Cat# 11965118
Penicillin-Streptomycin (P/S)	Thermo Fisher Scientific	Cat# 15140122
KnockOut™ DMEM	Thermo Fisher Scientific	Cat# 10829018
Opti-MEM™ I Reduced Serum Medium	Thermo Fisher Scientific	Cat# 10829018

**Critical commercial assays**		

FastGene Gel/PCR Extraction Kit	Nippon Genetics	Cat# 91302
Lipofectamine 2000 Transfection Reagent	Thermo Fisher Scientific	Cat# 11668019
Corning® Matrigel® Growth Factor Reduced (GFR) Basement Membrane Matrix	Corning	Cat# 356231
Zyppy Plasmid Miniprep Kit	Zymo Research	Cat# D4020

**Deposited data**		

pX458-HypaCas9	https://www.addgene.org/Yuichiro_Miyaoka/	#190604

**Experimental models: Cell lines**		

HEK293T cells	RIKEN BioResource Research Center	RCB2202

**Oligonucleotides**		

Single strand DNA donors for RBM20 gene: ACAGATATGGCCCAGAAAGGCCGCGGT CTAGTAGTCCGGTGAGCCGGTCACTCTCCCCGA	Miyaoka et al.^7^ Kato-Inui et al.^8^	N/A
Oligonucleotides for gRNA (FW): CACCGGTCTCGTAGTCCGGTGAGC	Miyaoka et al.^7^ Kato-Inui et al.^8^	N/A
Oligonucleotides for gRNA (RV) AAACGCTCACCGGACTACGAGACC	Miyaoka et al.^7^ Kato-Inui et al.^8^	N/A
Forword primer for genomic PCR and Sanger Sequence in RBM20 gene TGTGTGGTTCTGTAGAGTTGGGAGT	In this paper	N/A
Reverse primer for genomic PCR and Sanger Sequence in RBM20 gene ATCCTGTCCCTCTTGTCATCTCC	In this paper	N/A

**Recombinant DNA**		

pX458-HypaCas9 plasmid	Takahashi et al.^1^ https://www.addgene.org/Yuichiro_Miyaoka/	Addgene #190604

**Software and algorithms**		

Cas-Designer	Park et al.^14^	http://www.rgenome.net/cas-designer/
CRISPRdirect	Naito et al.^15^	http://crispr.dbcls.jp/

**Other**		

C1000 Touch Thermal Cycler	Bio-Rad	1851197JB
On-chip SPiS	On-chip Biotechnologies	70001
On-chip Sort	On-chip Biotechnologies	N/A
On-chip T-Buffer	On-chip Biotechnologies	Cat# 2001014
2D Chip-Z1001	On-chip Biotechnologies	Cat# 1002004
Dispensing pipette tips for On-chip SPiS (sterile)	On-chip Biotechnologies	Cat# 1007001
SAPPHIRE LOW-RETENTION PIPETTE TIPS	Greiner Bio-One	Cat# 738259
Flat-Base Self-standing Tube 5m	Sarstedt	Cat# 60.9921.530

## Materials and equipment

### Cell culture medium

**Table:** Growth medium

Reagent	Final concentration	Amount
DMEM	1x	500 mL
FBS	10%	50 mL
Pen/Strep	1%	5 mL
**Total**	**N/A**	**555 mL**

Store at 4°C for up to 2 months.

**Table:** Growth medium 2

Reagent	Final concentration	Amount
DMEM	1x	500 mL
FBS	20%	100 mL
Pen/Strep	1%	5 mL
**Total**	**N/A**	**605 mL**

Store at 4°C for up to 2 months.

### Solutions

**Table:** Matrigel solution for thin coating

Reagent	Final concentration	Amount
KnockOut DMEM	1x	50 mL
Matrigel (GFR)	80 μg/ mL	500 μL
**Total**	**N/A**	**50.5 mL**

All reagents should be mixed on ice. Store at 4°C for up to 2 months.

### Other solutions

**Table:** 

Name	Reagents
SOC medium	2% Tryptone, 0.5% Yeast Extract, 10 mM NaCl, 2.5 mM KCl, 10 mM MgCl_2_, 10 mM MgSO_4_, 20 mM Glucose
LB-Ampicillin	1% Tryptone, 0.5% Yeast Extract, 1% NaCl, 100 μg/mL Ampicillin
LB-Ampicillin plate	1% Tryptone, 0.5% Yeast Extract, 1% NaCl, 1.5% Agar, 100 μg/mL Ampicillin
PBS	137 mM NaCl, 2.7 mM KCl, 10 mM Na_2_HPO_4_, 1.8 mM KH_2_PO_4_, pH adjusted to 7.4

## Step-by-step method details

### Cell transfection

**Timing: 2 days**

This section describes cell seeding and transfection. In this example, the described amounts of reagents are for single-cell dispensing in four 96-well plates. We also use pCAG-EGFP plasmid as a positive control for transfection (use your preferred reporter expression plasmid; we recommend one that strongly drives EGFP by the CAG promoter). Untreated cells are used as a negative control for transfection as well as genome editing. HEK293T cells in 1 well of a 12-well plate are typically dispensed into two 96-well plates. Below, we describe an example transfection procedure including positive and negative controls to perform single-cell dispensing into four 96-well plates (see below Table).

**Table:** Transfection in 12-well plate (For single-cell dispensing into four 96-well plates)

Well	DNA	Note
A1	px458-HypaCas9 plasmid and donor DNA	For single-cell dispensing into the first and second 96-well plates
A2	px458-HypaCas9 plasmid and donor DNA	For single-cell dispensing into the third and fourth 96-well plates
A3	pCAG-EGFP plasmid	For positive control of transfection
A4	Non-treated	For negative control of transfection and sorting

1. Cell seeding.
  a. Cover a single well of a 12-well plate with 500 μL of Matrigel solution.
    ***Note:*** We coat culture plates with Matrigel to improve the cell attachment.
  b. Incubate the 12-well plate at 37°C for at least 1 h to coat the plate.
  c. Detach HEK293T cells with 0.25% Trypsin-EDTA, and then count the cell number.
  d. Remove Matrigel solution from the 12-well plate, seed HEK293T cells at 2×10^5^ cells/well, and then incubate in a 37°C 5% CO_2_ incubator overnight (12–24 h).
2. Transfection of px458-HypaCas9 plasmid with donor DNA.
  a. Adjust the DNA concentration of px458-HypaCas9 in water to 1,000 ng/μL.
  b. Adjust the donor DNA concentration to 100 ng/μL.
  c. Prepare Mixture 1 and Mixture 2 by pipetting as shown in (see below Tables).
  d. Add 40 μL/well of Mixture 1 40 μL/well of Mixture 2, mix well, and then incubate at room temperature for 20 min.
    **Table:** Mixture 1 (for 1 well)

Reagent	Final concentration	Amount
Opit-MEM	N/A	40 μL
Lipofectamine 2000	2.4 μL/well	2.4 μL
**Total**	**N/A**	**42.4 μL**
    **Table:** Mixture 2 (for 1 well)

Reagent	Final concentration	Amount
Opit-MEM	N/A	40 μL
px458-HypaCas9	720 ng/well	7.2 μL
Single-stranded donor DNA	80 ng/well	0.8 μL
**Total**	**800 ng/well**	**48 μL**
  e. Drip 80 μL/well of the incubated solution onto HEK293T cells in a 12-well plate.
  f. Observe the fluorescence of the transfected wells the next day.
    **CRITICAL:** Transfection is an important factor in deciding the success or failure of single-cell dispensing. If cells are damaged during transfection, cloning efficiency will go down.
    ***Note:*** This transfection protocol works for HEK293T and HeLa cells. We recommend using Lipofectamine 3000 for transfection of PC9 cells.^1^
    ***Alternatives:*** Other common lipofection reagents can be used for transfection. Lentiviral gene delivery can also potentially be used.

3. Coating a 96-well plate with Matrigel.
  a. Add 30 μL/well of Matrigel solution to a 96-well plate.
  b. Incubate the plate in a 37°C 5% CO_2_ incubator overnight (3–24 h).

***Note:*** We recommend preparing Matrigel-coated 96-well plates the day before dispensing single cells.

***Alternatives:*** Coating with substances other than Matrigel is also viable (e.g., iMatrix-511).

### Cell soring with on-chip sort

**Timing: 30 min (for steps 4 and 5)**

This section describes a fast and minimum-damage cell sorting using On-chip Sort (On-chip Biotechnologies Co., Ltd., Tokyo, Japan), a cell sorter based on microfluidic chip technology.^13^ This process collects EGFP-positive cells, allowing only cells expressing HypaCas9 to proceed to the single-cell dispensing step.

***Note:*** We recommend optimization of the sorting conditions before actually proceeding to single-cell dispensing. In this protocol, only cells with a strong fluorescence intensity are collected to make sure that these cells express Cas9.

4. Collecting cells.
  a. Remove the growth medium and wash the cells once with 1 mL PBS.
  b. Detach the HEK293T cells from a 12-well plate with 200 μL/well of 0.25% Trypsin-EDTA for 3 min at 37°C.
  c. Add 800 μL/well of growth medium and resuspend the cells by gentle pipetting.
  d. Collect the cell suspension in a 1.5-mL tube and centrifuge at 280 × *g* for 3 min at room temperature.
  e. Remove the supernatant by a pipette and add 350 μL of fresh growth medium to resuspend the cell pellet.

***Optional:*** In step 4e, genome editing efficiency can be estimated from EGFP-positive cells enriched with sorting by collecting 50 μL of cell suspension.

**Figure 2:**
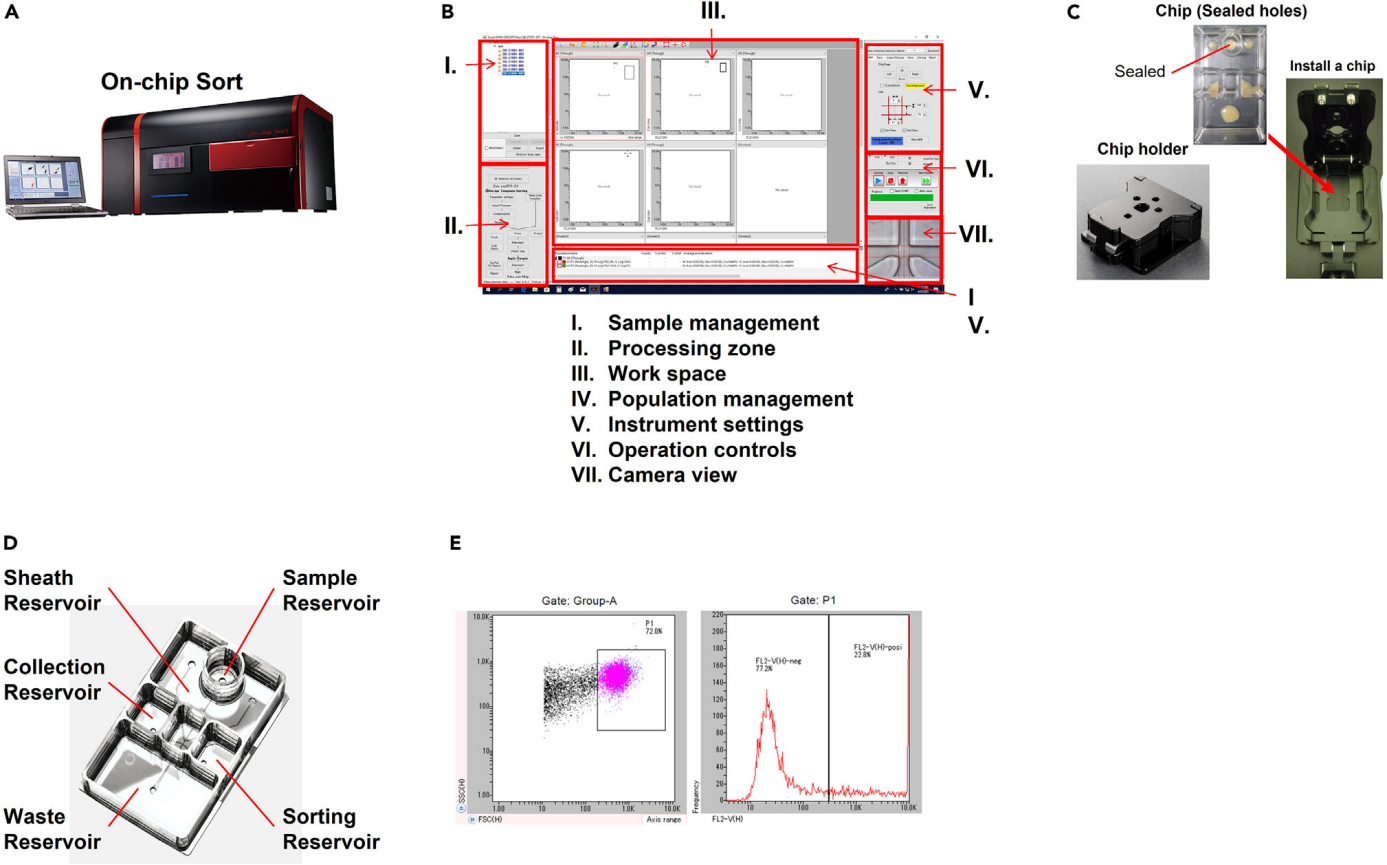
On-chip Sort system with microfluidic chips and sorting plots (A) Main unit of the On-chip Sort system. (B) On-chip Sort system operation screen. (C) Sealed holes chip for microfluidic adjustment and chip holder. (D) Microfluidic chip for On-chip Sort. (E) Sorting plot of HEK293T cells transfected with the px458-HypaCas9 plasmid. The left and right panels show the sorting gate for FSC vs. SSC and the histogram for FL2-V (EGFP), respectively.
    ii. Select the “OnChipFlow/Sort” icon to start the sorting control software program (Figure 2B).
    iii. After logging in, create a sample sheet.
    iv. Set the accuracy adjustment chip (sealed hole) shown in Figure 2C in the chip holder and insert it into the main sorter unit.
    v. Perform “Auto Check line” with an adjustment chip to carry out fine tuning to maintain sorting accuracy.
    vi. When a dialog box appears, select “OK”.
  b. Cell sorting.
    i. Set the new chip in the chip holder, add the following amount of sheath liquid (On-chip T-Buffer) to each reservoir (Figure 2D), and insert it into the main sorter unit:
      Sheath Reservoir: 8.0 mL.
      Sorting Reservoir: 1.7 mL.
      Collection Reservoir: 500 μL.
      Sample Reservoir: 500 μL.
    ii. Select "Prime" in the “Operation controls” panel to clean the microfluidic lines.
    iii. Remove the sheath liquid from the sample reservoir as well as most of the sheath liquid from the collection reservoir, leaving about 50 μL of it.
    iv. Transfer 150 μL of cell suspension prepared from step 4e to a new 1.5-mL tube and add 50 μL of T-Buffer.
    v. Transfer the full volume of the cell suspension prepared in the previous step (step 5b - (4) to the sample reservoir.
    vi. Set the chip holder containing the cell suspension into the main sorter unit.
    vii. Select "Run" in the “Operation controls” panel to start pre-sorting.
    viii. Sort for about 10–30 s, and then select “Stop”.
    ix. Check the plots displayed in "Work space" and identify the cell population you intend to recover by gating it.
      ***Note:*** Our typical gate settings for sorting EGFP-positive HEK293T cells are shown in Figure 2E. You can analyze non-treated cells to confirm proper gate settings. The upper limit of the optimal sorting rate is 30 events/sec; if the number of events is too high, the sample should be diluted with T-Buffer to achieve 30 events/sec.
    x. After setting up the gates, select "Sorting" from the "Operation Control" panel.
    xi. After collecting 2,000 cells, transfer them to a new 1.5-mL tube, and then add T-Buffer up to 300 μL.
      ***Note:*** The cell density of the suspension (2,000 cells/300 μL T-Buffer) collected in step 5b - xi has been optimized for the next "automatic dilution" step with the SPiS.

5. Cell sorting.
  a. Cell sorter setup.
    i. Turn on the sorter and a connected computer (Figure 2A).

### Single-cell dispensing with on-chip SPiS

**Timing: 2**–**3 h (for steps 6 to 10)**

**Figure 3:**
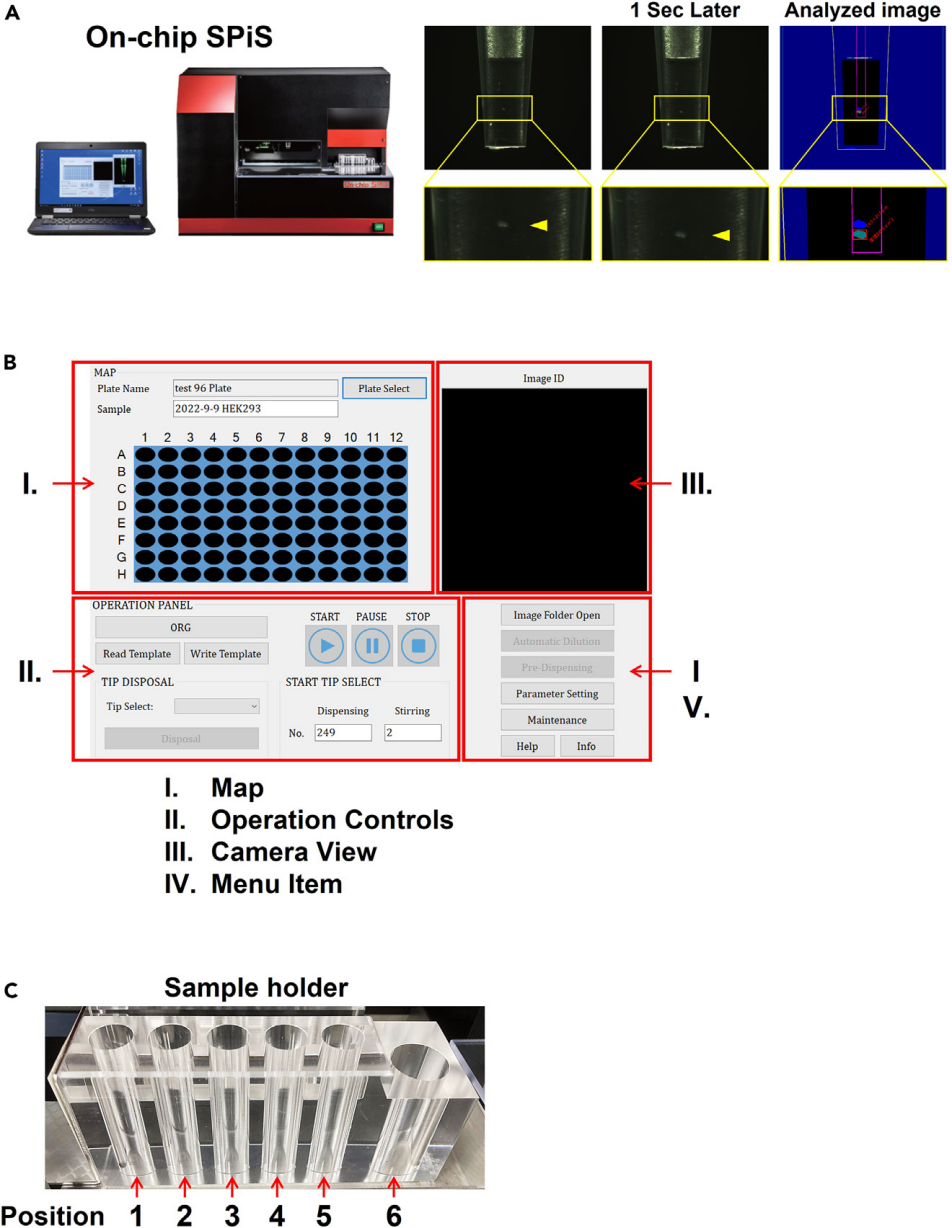
Results of an image analysis for single-cell dispensing by the SPiS and the operation screen of the system (A) The left panel shows the main unit of the SPiS. The right panel shows the image recognition of cell suspension by the SPiS. The two images of the aspirated cell suspension are taken at 1-s intervals; if the SPiS recognizes a single cell, the cell is dispensed; if not, the SPiS aspirates the cell suspension again, and the process is repeated. (B) The On-chip SPiS operation screen. (C) Sample holder in the SPiS. Note that only position 6 is equipped with 5-mL tubes.

This section describes fast and efficient single-cell dispensing using the SPiS. The SPiS is an automated single-cell dispenser that can gently and accurately dispense single cells into multi-well plates using image recognition technology to monitor the number of cells in aliquots (Figure 3A). In this protocol, single-cell dispensing with the SPiS is performed after sorting cells with On-chip Sort as described above. However, cell suspension prepared by other pretreatments (e.g., sorting by BD FACSAria III) can also be used. With FACSAria III, for example, suspension of sorted cells should be prepared to be as 2,000 cells in 300 μL T-buffer in a 1.5-mL tube. The cell suspension can be used directly in step 7a below.

6. The SPiS setup.
  a. Turn on the SPiS unit and a connected computer (Figure 3A).
  b. Launch the software program to control the SPiS (Figure 3B).
  c. Input the information of the plate into which cells are dispensed (Figure 3B).
  d. Select "ORG" in the operation screen panel and set the pipetting system at the initial position in the SPiS.
7. Perform “Automatic dilution” with the SPiS.
  a. Set the following tubes in the sample holder (Figure 3C):
    Position 1: 1.5-mL tube containing cell suspension from step 5b - xi.
    Position 3: 1.5-mL tube containing 300 μL of PBS (for Waste)
    Position 5: 1.5 mL empty tube (for dilution)
    Position 6: 5 mL flat bottom tube containing 5 mL of PBS.
  b. Select "Automatic dilution” in the “Menu Item”.
  c. Enter the settings below and select Start.
    Number of Times: 10.
    Number of Cells: 1.
    Histogram Class: 100.
  d. Check the histogram and select "Proceed". The diluted cell suspension is prepared in an empty tube at position 5.
  e. When the operation is complete, remove the tube in positions 1.
  f. Transfer the tube in position 5 to position 1.
8. Pre-dispensing with the SPiS.
  a. Remove the Matrigel from the 96-well plate (step 3b) and put it in the plate holder of the SPiS.
  b. Select “Pre Dispensing” in the “operation panel” and dispense 30 μL of PBS into the 96-well plate.

***Note:*** The SPiS dispenses single cells into solution, so PBS must be pre-dispensed into a 96-well plate. step 8b can be skipped by manually pre-dispensing PBS into a 96-well plate.

9. Single-cell dispensing with the SPiS.
  a. From the menu screen, select "START" to start dispensing.
    ***Note:*** The dispensing operation takes about 1 h for a 96-well plate.
  b. After finishing dispensing into the first plate, set a second 96-well plate and start the dispensing operation in the same manner from step 8b.
  c. Start preparing the third and fourth 96-well plates while the single-cell dispensing into the second 96-well plate is ongoing. Return to step 4 and repeat cell sorting and single-cell dispensing.
    ***Note:*** Two 96-well plates per tube of cell suspension are the maximum number viable for efficient single-cell dispensing. Dispensing more than two plates may cause cells to drop down and/or attach to the bottom of the sample tube, which slows down the dispensing speed.

10. Addition of conditioned medium and cell culture.
  a. After single-cell dispensing, add 100 μL/well of conditioned medium pre-warmed to 37°C using a multichannel pipette.
  b. The culture medium should be replaced with growth medium every week.
  c. Two weeks after cell dispension, remove the medium in 96-well plates and wash once with 100 μL/well of PBS, detach the survived cells in the 96-well plate with 10 μL/well of 0.25% Trypsin-EDTA.
    ***Note:*** The purpose of step 10c is to disperse the cells in a well to give them enough space for growth.
  d. Add 100 μL of growth medium 2 and resuspend cells in the same plate.
    ***Note:*** Trypsin is neutralized by FBS so that the cells can be cultured after step 10d without any further treatments.
  e. Keep culturing the cells until they become confluent.
    ***Note:*** The established clones can be transferred into 12- or 6-well plates for expanded culture. Genomic DNA can be extracted from a portion of the detached cells when the clones are transferred to expanded culture. Genomic DNA extraction can be performed by ethanol precipitation after overnight (12–72 h) incubation at 55°C in cell lysis buffer (10 mM Tris pH7.5, 10 mM EDTA, 10 mM NaCl, 0.5% N-Lauroylsarcosine, 0.1 mg/mL Proteinase K),^7^ or the rapid extraction method with Alkaline lysis solution (HoTSHOT method).^20^ The genome editing results of clones can be analyze by common polymerase chain reaction (PCR) and/or Sanger sequencing. We perform multiplexed amplicon sequencing to genotype many clones (over a hundred clones).^1^^,^^21^ Note that most of the cultured cell lines do not have a stable chromosome number and carry more than two copies of genes.

## Expected outcomes

**Figure 4:**
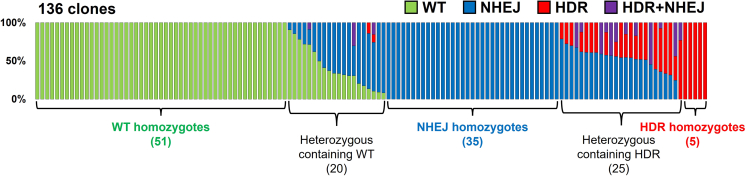
Example of genome-edited cell clones with various genotypes obtained by this protocol Genome editing outcomes in *RBM20* in individual HEK293T cells are shown. The respective bars in the Y-axis show the clones, and the X-axis shows the allele frequencies. The R636S point mutation was introduced by HDR induced by HypaCas9. However, NHEJ simultaneously induced insertions and deletions as byproducts. The genotypes of these clones were identified by amplicon sequencing.

Following this protocol, over 80 clones of genome-edited HEK293T cells can be established using four 96-well plates (Figure 4). For example, when a point mutation (R636S) was introduced into the *RBM20* gene in HEK293T, out of obtained 136 clones, 85 (62.5%) had some kinds of editing occurred, and 35 (25.7%) and 5 (3.7%) of those 85 clones were homozygous for NHEJ and HDR, respectively.^1^ The editing efficiency is dependent on the editing tool used. Base Editing^9^^,^^10^^,^^11^ and Prime Editing^12^ may improve the genome editing efficiency, especially in replacement of nucleotides. The use of longer donor DNA strands may also improve the efficiency.

## Limitations

This protocol is optimized for HEK293T cells, HeLa cells, and PC9 cells; it may therefore need to be optimized for other cell lines. The damage to the cells induced by transfection causes inefficient clone isolation. In fact, we experienced successful single-cell dispensing of some cell lines when non-transfected, but the cloning efficiency is significantly reduced when the same cell lines are transfected (e.g., HT-1080 cells and U2OS cells). In these cases, the clone recovery efficiency may be improved by reducing the damage induced by transfection. However, these milder conditions may compromise the transfection efficiency, so the transfection protocol needs to be carefully optimized. In addition, it is difficult to apply this protocol to cells prone to apoptosis when singularized, such as human pluripotent stem cells.

## Troubleshooting

### Problem 1

Low cloning efficiency due to cell line characteristics.

### Potential solution

Dispensation of untreated cells and adjustment of culture conditions, depending on cell line characteristics. Since different cell lines have different adhesion and proliferation abilities, single-cell cloning can be difficult, depending on cell line characteristics. Performing single-cell dispensing of untreated (regularly maintained without transfection or any treatments) cells by the SPiS can clarify whether or not it is possible to apply this protocol to that cell line. In addition, changing the culture medium and/or the coating material may improve the cloning efficiency.

### Problem 2

Low cloning efficiency due to transfection damage to cells (related to step 2).

### Potential solution

Damage to cells caused by transfection is an important determinant of the success of this protocol. If cell damage is suspected by microscopic observation of the cells or other ways, the transfection conditions should be revised. Easing the transfection conditions, such as by reducing transfection reagents, may improve the cell viability. However, keep in mind that easing the transfection conditions too much may result in inefficient genome editing.

An alternative solution is to continue culturing the cells after transfection for one to two weeks to allow them to recover from the damage. We cultured HEK293T cells that had been targeted to genome edit *RBM20* for about one month with regular passaging and confirmed that the frequencies of the edited *RBM20* alleles stayed basically constant. Therefore, longer culture would still allow us to isolate cells with intended genotypes in some cases while minimizing the effects of cell damage. However, this strategy will not work for genes that affect the cell proliferation and/or survival.

### Problem 3

Low cloning efficiency due to delayed single-cell dispensing with the SPiS (related to step 9).

### Potential solution

It is critical to avoid any contaminants or debris during single-cell dispensing. The accuracy of single-cell dispensing by the SPiS is ensured by image recognition of the cell suspension aspirated into a tip by a CCD camera. If the SPiS recognizes more objects other than a single cell, it will aspirate a new aliquot of the cell suspension (Figure 3A). Therefore, if the original cell suspension contains substantial contaminants or debris, the number of aspiration and image recognition trials by the SPiS will increase significantly, thereby leading to longer operating times for cell dispensing and increased cell damage, which results in a reduced cloning efficiency. This problem can be solved by filtering the media and solutions used in this protocol. In addition, be dedicated to neat and clean operations to avoid contamination.

### Problem 4

Low editing efficiency.

### Potential solution

With this protocol, we usually observe that more than half of isolated HEK293T cell clones have some edits in the genome (Figure 4). If the edited clones are rare, extract genomic DNA from the sorted cell s to monitor pooled editing results by Sanger sequencing. If there is no or little editing, you should check the transfection conditions or redesign the gRNA.

### Problem 5

Variable growth speed of the clones (related to step 10e).

### Potential solution

In step 10e, some clones may grow very slowly or stop proliferating. Based on our experiences, such clones tend to die in expanded culture. Therefore, we recommend, to exclude such clones from the experiments.

## Resource availability

### Lead contact

Further information and requests for resources and reagents should be directed to and will be fulfilled by the lead contact, Yuichiro Miyaoka (miyaoka-yi@igakuken.or.jp).

### Materials availability

The px458-HypaCas9 plasmid (#190604) used in this protocol has been deposited to Addgene. All reagents and materials used in this manuscript are available upon request or prepared to be available from commercial sources. HEK293T cells are available from RIKEN BioResource Research Center (https://cell.brc.riken.jp/en/).

## Data Availability

This study did not generate or analyze datasets or code.
